# Manganese Exposure Enhances the Release of Misfolded α-Synuclein via Exosomes by Impairing Endosomal Trafficking and Protein Degradation Mechanisms

**DOI:** 10.3390/ijms252212207

**Published:** 2024-11-14

**Authors:** Dharmin Rokad, Dilshan S. Harischandra, Manikandan Samidurai, Yuan-Teng Chang, Jie Luo, Vivek Lawana, Souvarish Sarkar, Bharathi N. Palanisamy, Sireesha Manne, Dongsuk Kim, Gary Zenitsky, Huajun Jin, Vellareddy Anantharam, Auriel Willette, Arthi Kanthasamy, Anumantha G. Kanthasamy

**Affiliations:** 1 Parkinson’s Disorder Research Program, Iowa Center for Advanced Neurotoxicology, Department of Biomedical Sciences, Iowa State University, Ames, IA 50011, USA; rokaddd@gmail.com (D.R.); dilshan.harischandra@labcorp.com (D.S.H.); jie.luo@duke.edu (J.L.); viveklawana@gmail.com (V.L.); souvarish_sarkar@urmc.rochester.edu (S.S.); nive@iastate.edu (B.N.P.); mannesireesha@gmail.com (S.M.); dskim@iastate.edu (D.K.); 2 Isakson Center for Neurological Disease Research, Department of Physiology and Pharmacology, University of Georgia, 325 Riverbend Road, Athens, GA 30602, USA; ms82476@uga.edu (M.S.); yc02707@uga.edu (Y.-T.C.); gary.zenitsky@uga.edu (G.Z.); huajun.jin@uga.edu (H.J.); vellareddy.anantharam@uga.edu (V.A.); ak39563@uga.edu (A.K.); 3 Department of Neurology, Rutgers University, New Brunswick, NJ 07101, USA; auriel.a.willette@rutgers.edu

**Keywords:** neurotoxicity, manganese, alpha-synuclein, exosomes, Parkinson’s disease, endosomal recycling mechanisms, environmental factors, metals

## Abstract

Excessive exposure to manganese (Mn) increases the risk of chronic neurological diseases, including Parkinson’s disease (PD) and other related Parkinsonisms. Aggregated α-synuclein (αSyn), a hallmark of PD, can spread to neighboring cells by exosomal release from neurons. We previously discovered that Mn enhances its spread, triggering neuroinflammatory and neurodegenerative processes. To better understand the Mn-induced release of exosomal αSyn, we examined the effect of Mn on endosomal trafficking and misfolded protein degradation. Exposing MN9D dopaminergic neuronal cells stably expressing human wild-type (WT) αSyn to 300 μM Mn for 24 h significantly suppressed protein and mRNA expression of Rab11a, thereby downregulating endosomal recycling, forcing late endosomes to mature into multivesicular bodies (MVBs). Ectopic expression of WT Rab11a significantly mitigated exosome release, whereas ectopic mutant Rab11a (S25N) increased it. Our in vitro and in vivo studies reveal that Mn exposure upregulated (1) mRNA and protein levels of endosomal Rab27a, which mediates the fusion of MVBs with the plasma membrane; and (2) expression of the autophagosomal markers Beclin-1 and p62, but downregulated the lysosomal marker LAMP2, thereby impairing autophagolysosome formation as confirmed by LysoTracker, cathepsin, and acridine orange assays. Our novel findings demonstrate that Mn promotes the exosomal release of misfolded αSyn by impairing endosomal trafficking and protein degradation.

## 1. Introduction

A group of neurodegenerative diseases known as synucleinopathies is characterized by the presence of Lewy bodies and Lewy neurites, predominantly composed of misfolded α-synuclein (αSyn). Among these diseases, Parkinson’s disease (PD) remains the second most prevalent neurodegenerative disease. PD progressively damages dopaminergic neuronal somata located in the substantia nigra pars compacta (SNpc) and their axonal projections into the striatum, causing progressively worsening motor symptoms such as rigidity, movement slowness, uncontrollable tremor, and postural imbalance [1]. It is critical to understand how synucleinopathy spreads, given that pathological αSyn is present in human CSF and plasma [2,3,4] and that αSyn-positive Lewy bodies propagate in grafted neurons [5] and embryonic nigral transplants of PD subjects [6].

Alpha-Syn can be secreted into the extracellular milieu through an exosomal pathway [7,8,9,10]. First detected independently by Pan et al. [11] and Johnstone et al. [12] three decades ago, exosomes are nanoscale (30–150 nm) membrane vesicles, which are formed via the endosomal system and secreted upon binding of multivesicular bodies (MVBs) to the plasma membrane. Initially, exosomes were thought to function only as cellular detritus carriers. Later, exosomes were reported to have an immunological role [13]. Numerous studies have since shown that exosomes serve as cargo vesicles containing molecules, including proteins and miRNAs, critical to intercellular communication linked to cell proliferation, the immune response, and disease progression [14].

The cell-to-cell transmission of misfolded protein is increasingly recognized as a critical component of progressive neurological diseases [15,16,17,18]. Several models have been proposed, including receptor-mediated endocytosis, exocytosis, tunneling nanotubes, cell injury, and exosomes [16,19]. Aside from the genetic predisposition to familial PD, exposure to environmental factors such as certain metals, solvents, and pesticides can increase the risk of developing idiopathic PD [20,21]. Chemical toxicant-induced exosome release and subsequent response in exosomes’ recipient cells have been gaining much interest recently, specifically due to the different cargo molecules that exosomes carry [22,23,24,25].

Manganese (Mn) toxicity has been linked with neurological deficits [26,27,28,29,30,31,32]. Humans are exposed to Mn via numerous routes, including drinking water, pesticides, contaminated air, and occupational exposure to welding fumes, mining, and the manufacturing of dry batteries and steel [26,33]. Even though Mn is a vital micronutrient in trace amounts [34], overexposure can cause manganism, which is another extrapyramidal disease manifesting motor, cognitive, and psychological dysfunction similar to PD [35,36]. However, PD and manganism differ in some distinctive neurological symptoms, and manganism is non-responsive to levodopa [37,38]. Despite the studies linking Mn overexposure to an elevated risk of PD [26,39], as well as our recent findings that Mn increases the release of misfolded αSyn-containing exosomes from dopaminergic neurons and promotes αSyn’s cell-to-cell transmission [10], the underlying molecular signaling mechanisms remain unclear.

In the present study, we examined the hypothesis that Mn promotes the exosomal release of αSyn by modulating endosomal protein trafficking and αSyn degradation mechanisms. Our systematic approach examined both Rab11a and Rab27a, proteins from a group of small Ras-like GTPases that serve as multifaceted regulators of various membrane-trafficking mechanisms in eukaryotic cells. Rab11a is a well-characterized Rab GTPase associated with endosomal recycling, secretory vesicles, and the trans-Golgi membrane [40,41,42]. Rab27 has been well-conserved among metazoan GTPases, and of its two isoforms, Rab27a and Rab27b, we focused on Rab27a, which is involved in exosome release via the fusion of MVBs with the plasma membrane [43,44]. Under normal conditions, early endosomes are converted into late endosomes. Late endosomes then mature into MVBs by invagination of the endosomal membrane to form intraluminal vesicles [45]. These mature MVBs either merge with the plasma membrane to release their contents as exosomes into the extracellular milieu [46] or merge with lysosomes for lysosomal cargo degradation. Herein, we show that Mn exposure downregulates Rab11a and upregulates Rab27a, with both effects inducing exosomal release. Our study also revealed that Mn interferes with the autophagic lysosomal degradation of aggregated αSyn by inducing lysosomal dysfunction. Collectively, these novel findings suggest that Mn stimulates the exosomal release of misfolded αSyn by compromising the endosomal and autophagic/lysosomal pathways.

## 2. Results

### 2.1. Generation of GFP-Tagged MN9D Dopaminergic Cells Stably Expressing Human αSyn

**Figure 1:**
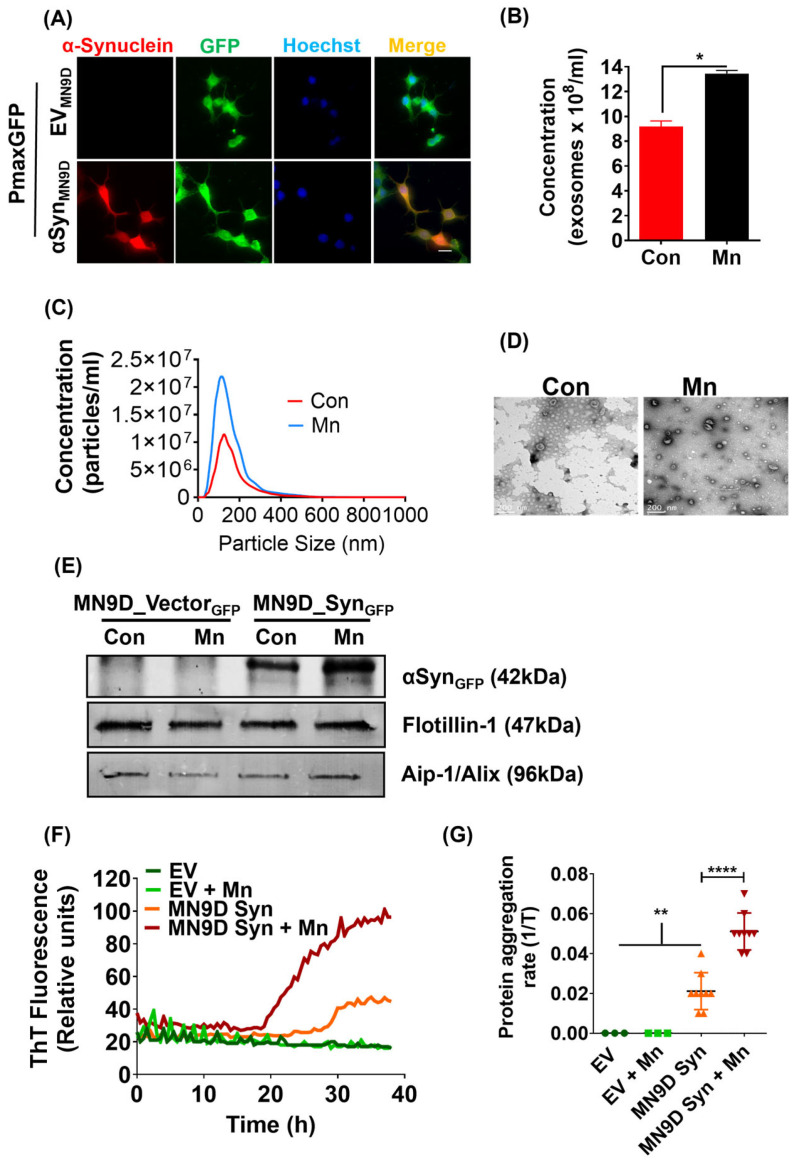
Generation of GFP-tagged MN9D dopaminergic cells stably expressing human αSyn and characterization of exosomes. (**A**) Immunofluorescence of stably expressed GFP-fused human αSyn (red) in MN9D_αSynGFP cells, and GFP fluorescence (green) in both vector (control) MN9D_EVGFP cells and human αSyn-expressing MN9D_αSynGFP cells. Nuclei were stained with Hoechst dye (blue). Magnification, 60×. Scale bar, 100 µm. (**B**) Nanoparticle tracking analysis showing the concentration of exosomes from MN9D_αSynGFP cells from vehicle-stimulated (red) and Mn-stimulated (black) cells. (**C**) Nanoparticle tracking analysis showing the size distribution of exosomes from MN9D_αSynGFP cells from vehicle- (red) and Mn-stimulated (blue) cells. (**D**) TEM image of exosomes secreted from MN9D_αSynGFP cells displays distinctive morphology. Scale bar, 200 nm. (**E**) Western blots of GFP-fused human αSyn in exosomes from MN9D_αSynGFP cells compared to MN9D_EVGFP cells relative to exosome-positive markers Flotillin-1 and AIP-1/Alix in both cell types. (**F**) Average Thioflavin T (ThT) fluorescence in exosome samples isolated from Mn-treated and untreated αSyn-expressing and EV_MN9D cells, showing more aggregated αSyn in Mn-treated αSyn-expressing cells. (**G**) Protein aggregation rates (PAR) in exosome samples. Each trace and dot represent the average of 4 technical replicates. Statistically significant differences are denoted as * *p* ≤ 0.05, ** *p* < 0.01, and **** *p* < 0.0001. *n* = 2–9.

We first utilized a transgenic neuronal cell model to dissect mechanisms underlying Mn-induced αSyn-containing exosomal release. To generate the MN9D_αSynGFP dopaminergic neuronal cells expressing human αSyn, we stably transfected MN9D cells with a construct encoding for N-terminal GFP-tagged WT human αSyn. The MN9D_EVGFP control cell line was also created through stable transfection of a pmaxFP-Green-N control vector. Results from ICC analysis revealed that more than 90% of the MN9D_αSynGFP cells were positive for human αSyn tagged with GFP, and all MN9D_EVGFP cells were positive for GFP (Figure 1A). As described in our publication [10], both stable cell lines had a low-level expression of endogenous mouse αSyn, while MN9D_αSynGFP cells robustly expressed GFP-tagged human αSyn.

### 2.2. Mn Exposure Enhances αSyn Secretion via Exosomes

Previously, we reported that Mn exposure enhances αSyn secretion via exosomes. Here, we first examined whether our generated human αSyn-expressing MN9D stable dopaminergic cells are a suitable model system for investigating the molecular mechanisms underlying Mn-induced αSyn exosomal release. For this, we treated our MN9D_αSynGFP cells with 300 μM of Mn for 24 h in serum-free media. The non-toxic Mn dose (300 µM) was chosen based on our results from an MTT cytotoxicity assay published previously [10] and other published studies [47,48]. This dose is also toxicologically relevant [10]. We collected the media and isolated exosomes using differential ultracentrifugation and counted exosomes using a NanoSight LM10 particle analyzer. Results from this experiment confirmed that Mn exposure significantly increased the release of exosomes (Figure 1B) in our MN9D_αSynGFP stable cells. With NanoSight analysis, we also confirmed that Mn exposure did not alter exosome size (Figure 1C) as we previously reported [10]. Morphologically, exosomes from conditioned media collected from Mn- and vehicle-treated MN9D_αSynGFP cells appeared via TEM to be oval-shaped vesicles (Figure 1D). Western blot analysis of exosomes isolated from both MN9D_EVGFP and MN9D_αSynGFP cells also confirmed the expression of the exosome marker proteins AIP-1/Alix and Flotillin-1 (Figure 1E). In line with our previous observations [10], we found predominantly more αSyn-GFP protein in the exosomes isolated from Mn-exposed cells than from untreated cells as determined by Western blot analysis (Figure 1E), confirming that Mn increases the amount of αSyn cargo in exosomes.

### 2.3. Determination of Misfolded αSyn Seeding Activity from Cell Culture Exosomes

The RT-QuIC assay was used to determine misfolded αSyn seeding activity from exosomes isolated from the Mn-treated and untreated MN9D_αSynGFP and MN9D_EVGFP cells by detecting kinetics of misfolded αSyn. Exosomes isolated from Mn-treated MN9D_αSynGFP cells showed higher αSyn seeding activity and PAR than untreated MN9D_αSynGFP cells (Figure 1F,G), whereas both Mn-treated and untreated MN9D_EVGFP cells lacked αSyn seeding activity and no measurable PAR. Collectively, these results indicate that Mn could enhance misfolded αSyn formation in dopaminergic neuronal cells (Figure 1G).

### 2.4. Mn Exposure Downregulates Rab11a Expression

**Figure 2:**
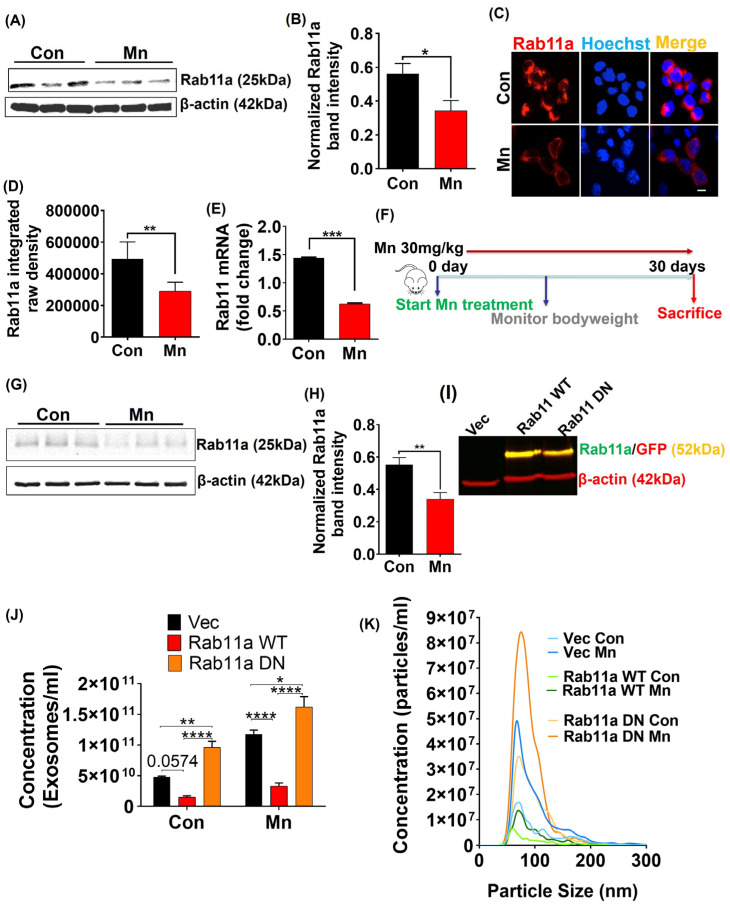
Mn exposure downregulates Rab11a expression both in vitro and in vivo. (**A**) Western blot of Rab11a from MN9D_αSynGFP cells with and without Mn exposure for 24 h. (**B**) Densitometry analysis of Rab11a in Mn-exposed and control MN9D_αSynGFP cells. (**C**) ICC analysis of MN9D_αSynGFP cells (Rab11a; red) with and without Mn exposure for 24 h. Nuclei were stained with Hoechst dye (blue). Magnification, 60×. Scale bar, 50 µm. (**D**) Quantitative analysis of Rab11a integrated raw density from immunofluorescence analysis. (**E**) RT-qPCR analysis of *Rab11a* mRNA expression in MN9D_αSynGFP cells with and without Mn exposure for 24 h. (**F**) Schematic illustration of Mn exposure in mice (30 mg/kg, male Swiss Webster and C57BL/6 mice) via oral gavage for 30 d. (**G**) Western blot of Rab11a from substantia nigral tissues from vehicle-treated and Mn-treated mice (30 mg/kg, male Swiss Webster). (**H**) Densitometry of Rab11a from substantia nigral tissues of vehicle- and Mn-exposed mice. (**I**) Western blot of control MN9D_αSynGFP (Vec) cells compared to the ectopic expression of wild-type (WT) Rab11a and dominant negative (DN) mutant Rab11a, respectively, in MN9D_αSynGFP cells transfected with WT Rab11a and DN Rab11 plasmids. (**J**) Concentration of exosomes from control and Mn-stimulated MN9D_αSynGFP cells (Vec), MN9D_αSynGFP cells expressing WT Rab11a, and MN9D_αSynGFP cells expressing DN Rab11a. (**K**) Nanoparticle tracking analysis showing the size distribution of exosome samples from exosome count, control and Mn-stimulated MN9D_αSynGFP cells (Vec; blue), MN9D_αSynGFP cells expressing WT Rab11a (green) and MN9D_αSynGFP cells expressing DN Rab11a (orange). Each group is represented by the mean ± S.E.M. from ≥3 separate measurements from vehicle- or Mn-treated groups. Statistically significant differences are denoted as * *p* ≤ 0.05, ** *p* < 0.01, *** *p* < 0.001 and **** *p* < 0.0001. *n* = 2–7.

With several studies reporting Rab11a’s involvement in endosome recycling near the plasma membrane [49,50], we examined whether Mn promotes the release of αSyn-containing exosomes by modulating the Rab11a-dependent endosome recycling pathway. For this, we first determined if there is any change in Rab11a expression in MN9D_αSynGFP cells treated with 300 µM Mn for 24 h relative to non-treated MN9D_αSynGFP cells. Interestingly, Mn exposure significantly downregulated Rab11a protein expression as revealed by both Western blot (Figure 2A,B) and ICC analysis (Figure 2C,D). RT-qPCR analysis revealed that *Rab11a* transcripts were also downregulated by Mn treatment (Figure 2E). Next, we tested the effects of Mn exposure on Rab11a expression in an animal model. Male Swiss Webster and C57BL/6 mice were on a 30 d treatment regimen of either water or Mn (Figure 2F). Western blot analysis of the substantia nigra (SN) revealed a significant downregulation of Rab11a protein expression in the Mn-exposed groups (Figure 2G,H). These results raise the possibility that Mn impairs Rab11a-dependent endosome recycling, thereby facilitating exosomal release.

### 2.5. Ectopic Expression of WT Rab11a and DN Mutant Rab11a (S25N) in MN9D_αSyn Cells Alters Exosome Release

To further evaluate the role of Mn-induced Rab11a downregulation in exosomal release, we transfected MN9D_αSyn cells with WT Rab11a-GFP and DN mutant Rab11a (S25N)-GFP constructs. Our Western blot analysis of transfected MN9D_αSyn cells using both Rab11a and GFP antibodies confirmed the ectopic expression of Rab11a (Figure 2I) in green and GFP in red, showing a yellow-colored band corresponding to the Rab11a-GFP fusion protein. After confirming the ectopic expression of WT Rab11a and DN Rab11a, we exposed these cells to 300 µM Mn for 24 h in serum-free media. We then isolated exosomes through ExoQuick-TC reagent and comprehensively assessed their size and concentration through the NanoSight LM10. Overexpression of WT Rab11a attenuated exosomal release in both untreated and Mn-stimulated MN9D_αSyn cells (Figure 2J), whereas ectopic expression of DN Rab11a enhanced exosome release (Figure 2J). The average size range of isolated exosomes was between 40 and 150 nm (Figure 2K), which is consistent with published values [8,9,10]. These results collectively suggest that the enhanced release of αSyn-containing exosomes after Mn treatment may be attributed at least in part to Mn-induced Rab11a downregulation.

### 2.6. Mn Exposure Upregulates Rab27a Expression

**Figure 3:**
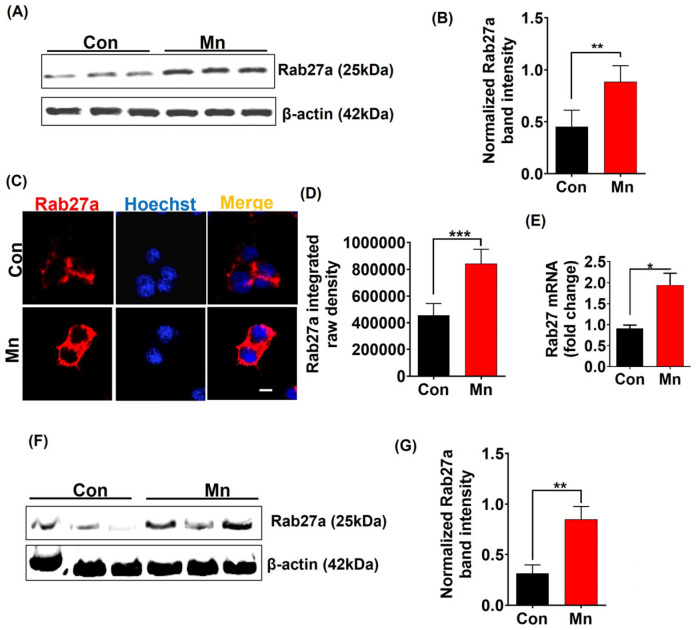
Mn exposure upregulates Rab27a expression both in vitro and in vivo. (**A**) Western blot of Rab27a in MN9D_αSynGFP cells with and without Mn exposure for 24 h. (**B**) Densitometry of Rab27a in Mn-exposed and control MN9D_αSynGFP cells. (**C**) Immunofluorescence analysis of MN9D_αSynGFP cells (Rab27a; red) with and without Mn exposure for 24 h. Nuclei were stained with Hoechst dye (blue). Magnification, 60×. Scale bar, 50 µm. (**D**) Quantitative analysis of Rab27a integrated raw density from immunofluorescence analysis. (**E**) RT-qPCR analysis of *Rab27a* mRNA expression in MN9D_αSynGFP cells treated with and without Mn exposure for 24 h. (**F**) Western blot of Rab27a from substantia nigral tissues from vehicle- and Mn-treated mice (30 mg/kg for 30 days, male Swiss Webster). (**G**) Densitometry of Rab27a in substantia nigral tissues of vehicle- and Mn-exposed mice. Each group is represented by the mean ± S.E.M. from ≥6 separate measurements from vehicle- and Mn-treated groups. Statistically significant differences are denoted as * *p* ≤ 0.05, ** *p* < 0.01, and *** *p* < 0.001. *n* = 4–10.

Rab27a reportedly helps regulate vesicular transportation and secretion [43,44], and therefore, we next examined the role of Rab27a during Mn-stimulated exosome release. Our Western blot, ICC, and RT-qPCR analyses revealed that exposing MN9D_αSynGFP cells to 300 µM Mn for 24 h significantly upregulated the expression levels of both Rab27a protein (Figure 3A–D) and mRNA (Figure 3E) in these cells. Importantly, the same 30 d treatment regimen of Mn also upregulated Rab27a protein expression significantly in the SN of male Swiss Webster and C57BL/6 mice (Figure 3F,G). These findings indicate that upregulation of the Rab27a-dependent trafficking and secretory mechanisms may play a role in Mn-induced exosome release.

### 2.7. Mn Exposure Impairs the Autophagic/Lysosomal System

**Figure 4:**
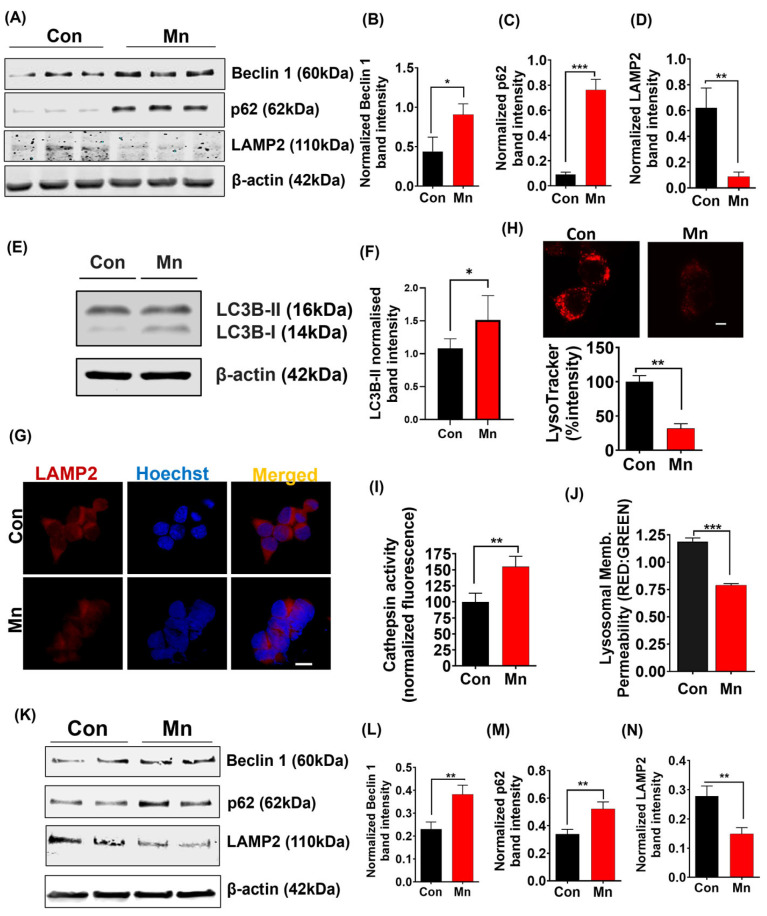
Mn exposure impairs the autophagic/lysosomal system in MN9D_αSynGFP cells. (**A**) Western blot of Beclin 1, p62, and LAMP2 from MN9D_αSynGFP cells with and without Mn exposure for 24 h. (**B**) Densitometry of Beclin 1 (**C**) p62 and (**D**) LAMP2 in vehicle- and Mn-exposed MN9D_αSynGFP cells. (**E**) Western blot of LC3B-I and II from MN9D_αSynGFP cells with and without Mn exposure for 24 h. (**F**) Densitometry of LC3B-II in vehicle- and Mn-exposed MN9D_αSynGFP cells. (**G**) Immunofluorescence analysis of MN9D_αSynGFP cells (LAMP2; red) with and without Mn exposure for 24 h. Nuclei were stained with Hoechst dye (blue). Magnification, 60×. Scale bar, 50 µm. (**H**) Immunofluorescence analysis showing reduced LysoTracker intensity in MN9D_αSynGFP cells following Mn treatment. Scale bar, 50 µm. (**I**) Cathepsin assay showing increased activity following Mn treatment. (**J**) Acridine orange assay showing Mn exposure reduced signal intensities. (**K**) Western blot analysis of lysosomal marker LAMP2 and autophagic markers Beclin 1 and p62 in the substantia nigra of mice with and without Mn exposure (30 mg/kg for 30 days, male Swiss Webster). (**L**) Densitometry of Beclin 1, (**M**) p62, and (**N**) LAMP2 in the substantia nigra of mice with and without Mn exposure. Statistically significant differences are denoted as * *p* ≤ 0.05, ** *p* < 0.01, and *** *p* < 0.001. *n* = 3–9.

**Figure 5:**
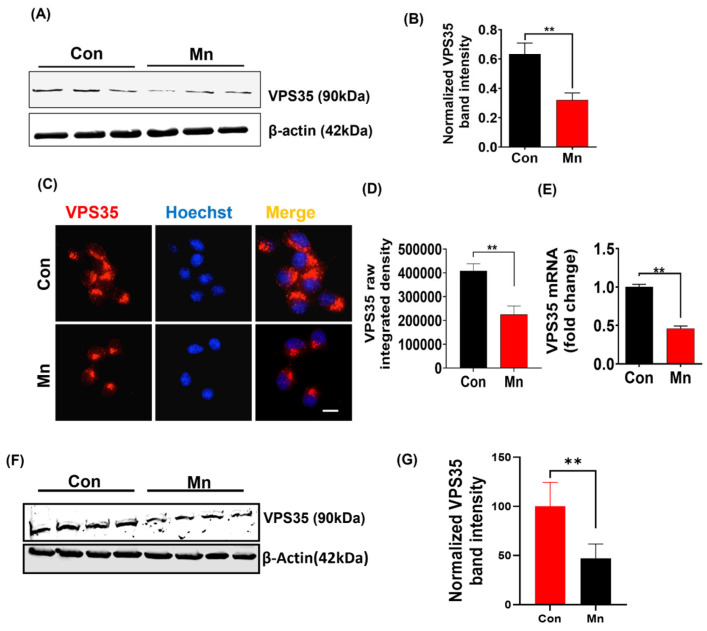
Mn exposure downregulates VPS35 expression in vitro. (**A**) Western blot of VPS35 from MN9D_αSynGFP cells with and without Mn exposure for 24 h. (**B**) Densitometry analysis of VPS35 in Mn-exposed and control MN9D_αSynGFP cells. (**C**) ICC analysis of MN9D_αSynGFP cells (VPS35; red) with and without Mn exposure for 24 h. Nuclei were stained with Hoechst dye (blue). Magnification, 60×. Scale bar 50 µm. (**D**) Quantitative analysis of VPS35 integrated raw density from immunofluorescence analysis. (**E**) RT-qPCR analysis of VPS35 mRNA expression in MN9D_αSynGFP cells with and without Mn exposure for 24 h. (**F**) Western blot analysis of VPS35 in the substantia nigra of mice with and without Mn exposure (30 mg/kg for 30 days, male Swiss Webster). (**G**) Densitometry of VPS35 in the substantia nigra of mice with and without Mn exposure. Statistically significant differences are denoted as ** *p* < 0.01. *n* = 2–8.

Misfolded proteins become degraded through the autophagic/lysosomal pathway [51], and Webb et al. [52] confirmed this for aggregate-prone αSyn protein. We treated MN9D_αSynGFP cells with 300 µM Mn for 24 h and then determined via Western blot analysis that Mn upregulated expression of the autophagosomal markers Beclin 1 and p62 (Figure 4A–C) but downregulated the lysosomal marker LAMP2 (Figure 4A,D). Supporting this, we also observed enhanced LC3B-I to LC3B-II conversion in Mn-treated cells (Figure 4E,F). Our ICC analysis also reveals that Mn downregulated LAMP2 expression (Figure 4G). To further confirm lysosomal dysfunction, we performed additional assays. Our LysoTracker assay shows that Mn exposure significantly reduced LysoTracker dye fluorescence (Figure 4H), while our cathepsin activity assay revealed that Mn exposure increased the release of this lysosomal enzyme indicating damaged lysosomes (Figure 4I), signifying lysosomal dysfunction following Mn exposure. We also performed an acridine orange assay in which the acridine orange dye can become trapped in healthy acidic lysosomes, whereas Mn exposure reduced the signal intensities suggesting damaged lysosomes (Figure 4J). Next, we tested the effects of Mn exposure on Beclin 1, P62, and LAMP2 expression in animal models (Mn-30 mg/kg for 30 days, male Swiss Webster and C57BL/6). Western blot analysis of SN tissues revealed a significant downregulation of LAMP2 protein expression (Figure 4K,N) and significant upregulation of Beclin 1 and p62 protein expression (Figure 4K–M) in the Mn-exposed group, confirming our results from in vitro studies. The vacuolar protein sorting 35 (VPS35, *PARK17*) is another protein that has gained significant interest in the field of PD research following its identification in a Swiss family with PD reported by Wider et al. [53]. VPS35 is reported to regulate lysosome function [54] by repeated retrieval of CI-MPR. CI-MPR is responsible for carrying newly synthesized hydrolase enzymes from the trans-Golgi network to endosomes and eventually to lysosomes. VPS35 interacts with the cytosolic domain of CI-MPR and sequesters it from endosomes, hence restricting its delivery to lysosomes where it can be degraded. We analyzed VPS35 following Mn exposure, and results revealed significantly downregulated levels of *VPS35* mRNA and protein (Figure 5A–E). Western blot analysis of SN tissues also revealed a significant downregulation of VPS35 protein expression in Mn-exposed animals (Figure 5F,G). We believe that Mn-induced downregulation of VPS35 may disrupt the sorting of acid hydrolyses and their receptor, such as CI-MPR, thereby leading to the receptor not being recycled properly and hydrolases not being sorted properly, which impairs the lysosomal function of degrading aggregated proteins such as αSyn. Collectively, these results suggest that Mn impairs autophagolysosome formation by damaging lysosomes and, thus, the autophagic/lysosomal pathway. Hence, Mn exposure promotes exosomal αSyn release by impairing intracellular αSyn clearance.

## 3. Discussion

It is being increasingly recognized that exogenous αSyn aggregates not only form Lewy body-like inclusions in recipient neuronal cells [9,55] but also trigger inflammatory and neurodegenerative responses in recipient microglial cells once released from neurons [56,57,58,59]. The addition of fibrillar αSyn to primary neurons, as well as neuronal cell models, recruits endogenous αSyn and converts it into detergent-insoluble, misfolded αSyn [17]. Parkinsonian symptoms can be generated in animal models of PD simply by introducing recombinant αSyn preformed fibrils or injecting pathogenic αSyn, suggesting that misfolded αSyn can act as a seed and/or template for the conversion of endogenous αSyn and the prionic spreading of αSyn aggregation [60,61,62].

It is now significantly clear that heavy metals such as Mn have a clear influential role when it comes to αSyn-related synucleopathies, specifically in the field of neurodegenerative diseases. Notably, recent findings indicate that Mn-induced αSyn overexpression suppresses Rab26-dependent autophagy in the presynaptic hippocampal neurons, leading to the accumulation of defective synaptic vehicles and synaptotoxicity, which ultimately contribute to neurological issues such as impairment of learning and memory [63]. These results, combined with our results from this study and previously published work from our lab [10,24,64,65,66], shed light on two crucial facts: (1) excessive exposure to Mn causes dysfunctions in a variety of cells across different regions of the brain, and (2) there is a clear connection between Mn and Rab proteins. While discussing heavy metals, other metals such as zinc, copper, and vanadium are also associated with an increased risk for neurological disorders such as PD [67,68]. A very crude research study looked at the effects of heavy metals such as zinc and copper on the human neuroblastoma cell line, SH-SY5Y. Interestingly, the study revealed that while these metals induce neuronal cell death, the molecular mechanisms involved differ from each other and from those induced by the well-known neurotoxin 6-hydroxydopamine (6-OHDA) [67]. While Mn toxicity had been shown in PD experimental models to trigger αSyn upregulation, aggregation, and neuronal apoptosis, we previously demonstrated that Mn exposure significantly increases the concentration of αSyn-packed exosomes enough to stimulate αSyn aggregation and propagation to neurotoxic levels [10].

The underlying key molecular mechanism by which Mn increases the release of exosomal αSyn remains unclear. Our work, presented here, began with a closer look at how Mn interacts with the exosome biogenesis and release pathways, specifically the Rab GTPases Rab11a and Rab27a, which are involved with endosomal trafficking and MVBs formation and fusion to the plasma membrane [46]. The MN9D_αSynGFP dopaminergic cell line we generated to stably express WT human αSyn clearly demonstrates that in vitro Mn exposure significantly downregulated the mRNA and protein expression levels of Rab11a, which is known to be associated primarily with recycling endosomes back to the membrane during endosomal trafficking [40,41,42,69]. Our in vitro findings regarding Mn-induced downregulation of Rab11a also extend to in vivo experiments where the effect was found in SN tissues. We also confirmed Rab11a specificity via ectopic expression experiments: in both untreated and Mn-stimulated MN9D_αSynGFP cells, ectopic expression of WT Rab11a attenuated exosomal release, whereas ectopic expression of DN mutant Rab11a (S25N) enhanced exosome release, strongly suggesting that Rab11a downregulation leads to a higher exosomal release. Mn-mediated downregulation of Rab11a suggests that Mn forces late endosomes to mature into MVBs, which occurs by inner membrane invagination. Once formed, MVBs can either fuse with the plasma membrane to release their contents outside of cells in the form of exosomes [45], or they can merge with lysosomes to degrade their contents through the autophagic lysosomal system [45,70].

Since our lab had previously demonstrated that Mn exposure significantly increases the number of exosomes [10], here we analyzed the fate of MVBs’ fusing with the plasma membrane, which ultimately releases exosomes into the extracellular space. This latter process is regulated by the GTPase Rab27a [43,44]. In contrast to our results for Rab11a downregulation, Mn exposure upregulated the expression levels of Rab27a in both in vitro and in vivo studies. This Mn-induced upregulation of Rab27a supports our hypothesis that Mn exposure downregulates Rab11a, forcing late endosomes to mature into MVBs, and upregulates Rab27a, and thus the merging of MVB cargo to the plasma membrane leading to the release of exosomes into the extracellular milieu.

Misfolded proteins become degraded when MVBs enter the autophagic lysosomal system by merging with lysosomes [46,70]. Thus, another logical step for us to test was the effect of Mn on the autophagic lysosomal system. We discovered that Mn exposure increases the autophagic response measured via autophagic markers Beclin 1 (autophagosome formation), p62 (linking cargo protein to autophagosome), and LC3B (protein lipidation process) while impairing the formation of autolysosomes by inducing lysosomal dysfunction analyzed by LAMP2 (lysosomal membrane protein), which can further contribute to the exosomal release of misfolded αSyn aggregates.

Recent studies have demonstrated that excessive exposure to and insufficient excretion of heavy metals, such as Mn, contributes to the development of neurological diseases. Several studies have shown that homeostatic mechanisms typically control metal levels in our body; however, any disruption to these delicate mechanisms can result in the accumulation of metals in our system. This accumulation and excessive exposure lead to neurological and other detrimental diseases [71,72,73]. Given these facts and the role of Mn in neurological conditions, it is important to understand the regulators of Mn in the body. Interestingly, recent research clearly identifies and proves that hypoxia-inducible factor (HIF) 1 and HIF2 are the key regulators of SLC30A10, a transporter protein that facilitates Mn excretion. These studies also showed that stabilizing HIF1 and HIF2 leads to the stabilization of SLC30A10, hence reducing Mn levels and consequently protecting cells and mice from Mn-induced toxicity [74,75,76]. Based on these findings, further studies are warranted to look at specific Mn regulators that can stabilize Mn levels in the body and, indeed, regulate the αSyn-exosomal pathway by facilitating proper regulation of Rab proteins and related downstream mechanisms.

**Figure 6:**
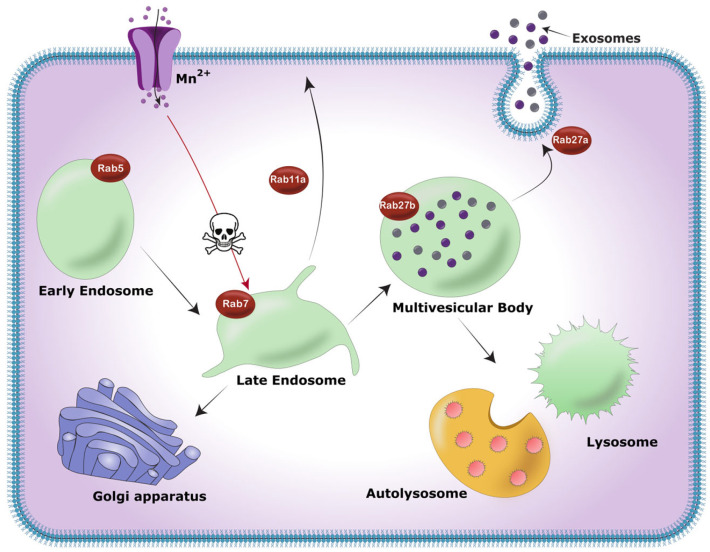
Schematic representation of Mn’s potential to disrupt the endosomal trafficking and lysosomal pathway. Under normal, stress-free conditions, early endosomes form and are converted into late endosomes. Late endosomes can either be recycled back to the plasma membrane, as mediated by Rab11a, or they can mature into multivesicular bodies. MVBs either fuse with the plasma membrane, as mediated by Rab27a, to release exosomes, or MVBs can also merge with a lysosome to degrade its contents through the autophagolysosome pathway. Our study supports a model in which, when cells are exposed to Mn, key mediators of this endosomal trafficking pathway, such as Rab11a and Rab27a, as well as the lysosomal pathway, become dysregulated, leading to increased exosome release. Rab5 and Rab7 are involved in the maturation of early endosomes to late endosomes. Certain graphical representations in the above schematic depict proteins, such as divalent metal-ion transporter-1 (DMT-1), which are involved in Mn transport and homeostasis and are shown here in the shape of ion channels. The skull markings represent toxic conditions from Mn exposure.

Collectively, results from this study suggest that Mn exposure forces the cell to release αSyn from the cell via exosomes instead of engaging in autophagic lysosomal degradation (Figure 6). Specifically, Mn forces late endosomes to mature into MVBs by downregulating Rab11a, instigating impairment of the autophagic lysosomal system, and causing the fusion of more MVBs to the plasma membrane by upregulating Rab27a, leading to the enhanced release of exosomes from the cells. These independent responses of Rab11a and Rab27a and lysosomal activity significantly increase exosomal αSyn release and the potential spread of neuropathology. Our results help to clarify the underlying mechanisms of Mn neurotoxicity through exosomal αSyn release and could help ongoing efforts to identify molecular targets for therapeutics and biomarker discovery.

## 4. Materials and Methods

### 4.1. Chemicals and Reagents

L-glutamine, fetal bovine serum (FBS), Lipofectamine 2000, Hoechst nuclear stain, Alexa fluorophore-tagged secondary antibodies, streptomycin, penicillin, and other cell culture reagents were obtained from Life Technologies (Gaithersburg, MD, USA). Mn chloride (MnCl_2_·4H_2_O), DMEM media, acridine orange, and all other chemicals were obtained from Sigma (St. Louis, MO, USA). Western blot materials such as gel-running units, nitrocellulose membranes, etc., and the Bradford protein assay kit were obtained from Bio-Rad Laboratories (Hercules, CA, USA).

### 4.2. Cell Culture, Treatment Paradigm and Ectopic Expression of Rab11a Variants

Cell culture was carried out as previously described in Harischandra et al. [66]. Briefly, MN9D cells stably expressing human αSyn-GFP fusion protein (MN9D_αSynGFP) were generated by transfecting MN9D cells with a pmaxGFP-αSyn fusion construct, while the control cell line (MN9D_EVGFP) was prepared by transfecting a pmaxGFP empty vector (EV) construct (Lonza, Basel, Switzerland). The stable cells were maintained in DMEM media supplemented with geneticin (200 μg/mL). Cells were treated at 70–80% confluency with 300 µM Mn for 24 h. Ectopic expression of WT Rab11a and dominant negative (DN) mutant Rab11a in MN9D_αSynGFP cells was carried out using Lipofectamine 2000 reagent (Invitrogen, Carlsbad, CA, USA) following the manufacturer’s protocol.

### 4.3. Western Blotting

RIPA buffer and protease and phosphatase inhibitor cocktail (Thermo Fisher Scientific, Waltham, MA, USA) were used to prepare whole-cell and tissue lysates. Equal amounts of proteins were separated using 12–15% as well as Any kD (Bio-Rad) precast SDS-polyacrylamide gels. Gels ran at 110 V for 1.5–2 h at 4 °C. After separation, proteins were transferred at 27 V overnight at 4 °C to nitrocellulose membranes (Bio-Rad/1620112) through electro-blotting. Non-specific sites on nitrocellulose membranes were blocked by incubating membranes with LI-COR blocking buffer. Primary antibodies for αSyn (Syn-1, BD Bioscience/610787, San Jose CA, USA), Flotilin-1 (Cell Signaling/18634, Danvers, MA, USA), GFP (Abcam/Ab184601, Cambridge, UK), Rab27a (Santa Cruz/SC-22756, Dallas, TX, USA), Rab11a (Cell Signaling/2413S), LAMP2 (Santa Cruz/sc-5571), Beclin1 (Cell Signaling/3495s), p62 (Cell Signaling/#5114), Alix (Millipore/ABC40, Burlington, MA, USA), LC3B (Cell Signaling/2775S), β-actin (Sigma/A5441), and VPS35 (Abcam/ab157220) were used to blot the membranes. IR800-conjugated anti-rabbit or Alexa Fluor 680-conjugated anti-mouse secondary antibodies were used to develop membranes. The Odyssey IR Imaging system (LI-COR/9120 and ODM-0128) and Odyssey 2.0 software were used for imaging and analyzing blots.

### 4.4. Immunocytochemistry (ICC)

After treatments, cells were fixed with 4% paraformaldehyde, washed with PBS, and blocked with 2% BSA, 0.05% Tween-20, and 0.5% Triton X-100 in PBS for 45 min. Cells were then incubated with primary antibodies overnight at 4 °C. After primary antibody incubation, cells were washed with PBS and incubated in the dark for 90 min with Alexa-488 and -555 dye-conjugated secondary antibodies (Invitrogen, 1:1000). Hoechst 44432 was used as a nuclear stain, and the coverslips were then mounted on glass slides and viewed with 63× and 43× oil objectives using a Leica DMIRE2 confocal microscope (Leica Microsystems, Wetzlar, Germany). Primary antibodies were obtained from the sources described for Western blotting.

### 4.5. RT-qPCR

RNA isolation and RT-qPCR were accomplished as previously described by Sarkar et al. [64]. Briefly, total RNA was isolated using the TRIzol reagent as per the manufacturer’s protocol, and concentration was measured using NanoDrop (Thermo Fisher Scientific, Waltham, MA, USA). First-strand cDNA synthesis was performed using an Affinity Script qPCR cDNA synthesis system (Agilent Technologies, Santa Clara, CA, USA). RT-qPCR was performed with the RT^2^ SYBR Green master mix (Thermo Fisher Scientific, Waltham, MA, USA). QuantiTect primers for the *Rab11a* and *Rab27a* genes (Qiagen, Germantown, MD, USA) were used for RT-qPCR. The housekeeping gene *18S* rRNA (Qiagen #PPM57735E) was used as the reference for all RT-qPCR experiments. Dissociation curves were run to ensure a single amplicon peak was obtained. The results are reported as fold change in gene expression, which was determined via the ΔΔCt method using the threshold cycle (Ct) value for the housekeeping gene and the respective gene of interest in each sample.

### 4.6. Exosome Isolation

Once the MN9D_αSynGFP and MN9D_EVGFP cells reached 70–80% confluency, they were treated with 300 μM Mn for 24 h. Conditioned media was then centrifuged at 300× *g* for 10 min to remove cell debris. The resulting supernatant was filtered using a 0.22-μm syringe filter (Millipore) to remove any remaining cell debris. After filtration, the filtrate was centrifuged at 100,000× *g* for 90 min using a Beckman Optima L-100 XP ultracentrifuge. The pellet containing exosomes was washed once with cold PBS and centrifuged again at 100,000× *g* for 90 min using a Beckman optima MAX ultracentrifuge (Beckman Coulter, Inc., Indianapolis, IN, USA). ExoQuick-TC was used according to the manufacturer’s protocol to isolate exosomes from the conditioned media in Rab11a ectopic expression experiments.

### 4.7. Nanoparticle Tracking Analysis (NTA)

After ultracentrifugation, exosomes were subjected to NTA, as previously described by Harischandra et al. [66]. Isolated exosomes were resuspended in approximately 1000 μL of sterile-filtered PBS. After resuspension, exosomes were further diluted into 500–1000 μL sterile-filtered PBS according to the exosome concentration. Around 300 μL was transferred to the sample chamber of the LM10 unit (NanoSight, Amesbury, UK) using a disposable syringe. Sample durations of 30–60 s per sample were analyzed with NTA 2.3 software (NanoSight). Samples containing higher numbers of exosomes were diluted before the analysis, and their relative concentrations were then calculated according to the dilution factor.

### 4.8. Real-Time Quaking-Induced Conversion (RT-QuIC)

The RT-QuIC assay was completed as published previously [10,77,78,79] using a 96-well clear bottom plate (Nalgene Nunc International, Rochester, NY, USA). For each RT-QuIC assay, recombinant human αSyn is used as a substrate, and exosomes isolated from the Mn-treated αSyn- or EV-expressing MN9D cells were used as a seed. In brief, 5 µL of the diluted exosomal sample was loaded along with 95 µL of the reaction mixture in each well of a 96-well plate preloaded with 6 silica beads 0.8 mm in diameter (OPS Diagnostics, Readington, NJ, USA). The RT-QuIC reaction mixture consists of final concentrations of 40 mM phosphate buffer (pH 8.0), 170 mM NaCl, 10 µM ThT, 0.0015% sodium dodecyl sulfate (SDS) and 0.1 mg/mL of recombinant αSyn. Recombinant αSyn was purified by expressing it in *E. coli* as described previously [77]. After loading the samples, plates were sealed with a plate sealer (Nalgene Nunc International), and ThT fluorescence readings were taken in a CLARIO star plate reader (BMG LABTECH, Cary, NC, USA) at excitation and emission wavelengths of 450 and 480 nm, respectively, every 30 min with alternating 1-min shake and rest cycles (double orbital, 400 rpm) at 42 °C. Samples were run in quadruplicates. Threshold fluorescence was calculated by taking the average fluorescence of the first 10 cycles for all samples plus 10 standard deviations. The protein aggregation rate (PAR) was calculated by taking the inverse of the time required to cross the threshold fluorescence.

### 4.9. Transmission Electron Microscopy (TEM)

Purified exosomes were resuspended in 200 mL PBS. We mixed 20 mL of each sample with uranyl acetate 2% (*w*/*v*), incubated them for 5 min, and then applied 5 mL to carbon-coated copper grids. Images were taken using a JEOL 2100 200 kV scanning (Peabody, MA, USA) and transmission electron microscope (STEM) with a NORAN System SIX elemental analysis system (Thermo Fisher Scientific, Waltham, MA, USA). TEM was operated at 80 kV, and images were obtained at 18,000–20,000× magnification.

### 4.10. Animal Studies

Male C57BL/6 and Swiss Webster mice (12–24 weeks old), obtained from Charles River and MMRRC, respectively, were used for all mouse experiments. Mice were housed on a 12-h light cycle with ad libitum access to food and water. To assess the impact of environmental Mn exposure on endosomal trafficking and protein degradation mechanisms, treatment group mice were exposed to Mn (MnCl_2_, 30 mg/kg) in Milli-Q water for 30 d via oral gavage while vehicle mice were similarly treated but with Milli-Q water alone. These Mn dose regimens were chosen based on previous studies in humans and animals [80,81,82]. Animals were euthanized using carbon dioxide (CO_2_) exposure in the chamber. Brains were dissected according to the previously published procedures [83]. Brain dissection and separation of midbrain regions were performed according to the previously published procedures [83,84,85]. Briefly, with the use of surgical scissors, mice were decapitated by cutting the skin along the midline on the dorsal side to open the skin barrier. The skull cap (Calcaria, Roman, UK) was removed by dissociating the sagittal suture. Using curved forceps, the brain was disconnected from the optic chiasm and the base of the head. Olfactory bulbs were removed using a sharp blade. Basal ganglia were accessed by removing the cerebral cortex. The substantia nigra was then meticulously separated from the striatum. Tissues were maintained on ice prior to storage at −80 °C.

### 4.11. Cathepsin Activity Assay

One million MN9D_αSynGFP cells were seeded in 6-well cell culture plates. Following a 24-h Mn treatment, cells were collected, and a cathepsin activity assay kit (Abcam) was used according to the manufacturer’s protocol. Briefly, cells were collected following the treatment and lysed with the kit’s cell lysis buffer. Dilutions were made, and intensities were measured on a plate reader according to the protocol provided in the kit.

### 4.12. Acridine Orange Assay

The acridine orange assay was performed as previously described [86]. MN9D_αSynGFP cells were seeded in 24-well plates. Following Mn treatment, cells were incubated with 5 µg/mL acridine orange solution for approximately 25 min in an incubator at 37 °C. Cells were washed with PBS. Excitation and emission intensities were measured at 480 nm and 529 nm, respectively, using a SpectraMax spectrophotometer (Molecular Devices Corporation, San Jose, CA, USA).

### 4.13. LysoTracker Assay

The LysoTracker assay (Thermo Scientific/L7528) was performed according to the manufacturer’s protocol. MN9D_αSynGFP cells were seeded in 24-well plates. Following Mn treatment, cells were incubated with a final concentration of 75 nM LysoTracker dye solution for approximately 1 h in an incubator at 37 °C. Cells were washed with PBS. Cells were then fixed with 4% PFA, mounted on glass slides, and viewed with 63× and 43× oil objectives using a Leica DMIRE2 confocal microscope.

### 4.14. Statistical Analysis

Statistical analysis was carried out using Prism 6.0 software for data from two or more individual experiments, each with *n* ≥ 6. One-way ANOVA was used for comparing multiple groups with Tukey post-analysis unless otherwise mentioned. For comparing two groups, Student’s *t*-test was used to find significant differences between treatment and control groups. Statistically significant differences were denoted as * *p* ≤ 0.05, ** *p* < 0.01, *** *p* < 0.001 and **** *p* < 0.0001.

## Data Availability

All data needed to evaluate the conclusions in the paper are present in the paper. Any material in this manuscript may be obtained through a material transfer agreement.
